# Causes and consequences of bacterial local adaptation via MGEs in the plant microbiome

**DOI:** 10.1111/nph.70766

**Published:** 2025-11-28

**Authors:** Stephanie Porter, Dominique Holtappels, Angeliqua Montoya, Britt Koskella

**Affiliations:** ^1^ School of Biological Sciences Washington State University Vancouver WA 98685 USA; ^2^ Plant Pathology and Plant‐Microbe Biology Section of the School of Integrative Plant Science Cornell University Cornell AgriTech Geneva 14456 NY USA; ^3^ Department of Integrative Biology University of California, Berkeley Berkeley 94720 CA USA; ^4^ Chen Zuckerberg Biohub Investigator San Francisco 94158 CA USA

**Keywords:** bacteria, genomic island, local adaptation, mobile genetic element, plant–microbe interactions, plasmid, prophage

## Abstract

Adaptations that enable plant‐associated bacteria to fill disparate niches comprise a critical component of microbial diversity. Genes that confer locally adaptive bacterial traits, ranging from heavy metal resistance to pathogen or symbiont infectivity, often reside within mobile genetic elements (MGEs) that can move between genomes. While MGEs may speed microbial adaptation, they also have selfish fitness interests and potentially separate evolutionary trajectories from their host genome. MGEs can also impose physiological burdens and be limited in the transmissibility of function across hosts, which likely constrains bacterial local adaptation. Given these constraints, the prevalence of adaptive loci on potentially exploitative MGEs poses a dilemma: how do fitness conflicts and alignments between MGEs and the main replicon shape bacterial local adaptation and impact plant hosts? We synthesize research on ways MGEs confer rapid, niche‐specific fitness advantages to bacteria, identify factors that promote or constrain bacterial adaptation, and highlight MGE impacts on plants. We focus on large, self‐transmissible MGEs (islands, plasmids, and prophages; though we expect relevance to other MGEs as well) to better understand how MGEs bolster yet constrain bacterial local adaptation. We specifically explore the role of MGEs in shaping bacteria that themselves play a role in expanding or contracting the plant niche.

**Table:** 

	Contents	
	Summary	2215
I.	Genomic architecture of local adaptation in plant‐associated bacteria	2215
II.	Fitness conflict and alignment during MGE‐mediated adaptation	2216
III.	The utility of MGEs for bacterial adaptation and consequences for host plants	2216
IV.	Costs of relying on MGEs for bacterial adaptation and consequences for plants	2220
V.	Conclusion: impact of MGEs on plant health	2221
	Acknowledgements	2221
	References	2221

## I. Genomic architecture of local adaptation in plant‐associated bacteria

Local adaptation, the greater performance of populations in local, compared with foreign conditions, measures the match between genetic and environmental variation (Wadgymar *et al*., 2022). In bacteria, loci conferring local adaptation can arise in a lineage via *de novo* mutation and lead to clonal sweeps (Arnold *et al*., 2022). By contrast, locally advantageous loci can spread across distinct bacterial lineages during gene‐specific sweeps due to gene flow via horizontal gene transfer (HGT) (Arnold *et al*., 2022). For example, in areas exposed to abiotic stresses, such as toxic heavy metals in the soil, belowground plant mutualistic bacteria can accumulate plasmids (Wright *et al*., 2024) and genomic islands (Kehlet‐Delgado *et al*., 2024) that bear loci enabling persistence. Similarly, pathogenic bacteria can adapt via plasmid‐borne antibiotic resistance loci, hampering efforts to maintain plant health (Verhaegen *et al*., 2023). Under biotic selection, soil bacteria can acquire genomic islands that enable them to infect novel host plants to benefit from mutualistic intracellular symbiosis (Weisberg *et al*., 2022), as well as mobile genetic elements (MGEs) that enhance pathogenic infectivity or virulence via effectors and other factors that interfere with plant defense (Pena *et al*., 2024).

The extent of local adaptation in plant‐associated bacteria will depend on the balance between the strength of local selection and gene flow among populations. As selection pressures shift across space or time, for example due to partner coevolution, local adaptation outcomes (i.e. the response to local selection) will be shaped primarily by mutation rates, cell migration, and/or HGT among lineages. The latter is mediated primarily by the transmission of MGEs, ranging from genomic islands, integrative and conjugative elements, and prophages embedded in the chromosome, to extrachromosomal circularized plasmids of various sizes and copy number (Box 1). Such MGEs can carry cargo genes such as accessory loci that bolster bacterial local adaptation by altering niche‐specific bacterial fitness (Vos *et al*., 2024). However, adaptation could also be constrained if the transmission or successful transfer of cargo gene function is limited across asexually reproducing lineages, or if HGT is so frequent that locally optimal genotypes are swamped by input of nonbeneficial ones (Stevenson *et al*., 2017; Maddamsetti & Lenski, 2018).

Box 1 **Mobile genetic elements**

The mobilome, or ecosystem of mobile genetic elements (MGEs), potentiates bacterial local adaptation by transmitting niche‐expanding genes among divergent lineages, although MGEs can also be exploitative (Weisberg & Chang, 2023). We highlight major classes of large MGEs (a–c), although MGEs comprise a spectrum of interrelated elements that includes intermediate forms (Lang *et al*., 2025).

**(a) Plasmids:** Circular or linear extrachromosomal elements that encode their own replication and maintenance. Plasmids are usually maintained at multiple copies per cell, producing islands of polyploidy in the genome (Rodríguez‐Beltrán *et al*., 2021). Conjugative plasmids encode the machinery to enable horizontal plasmid transmission to other cells, although plasmids can instead be mobilizable, and gain mobility via interactions with other MGEs within a cell, or be nonmobilizable (Brockhurst & Harrison, 2022). Thus, plasmids may transmit horizontally among lineages and/or vertically to the host's daughter cells.

**Genomic islands (GIs):** Horizontally acquired clusters of genes that integrate into the main replicon and can replicate via vertical transmission to daughter cells or horizontally among lineages if the GI can transmit between host lineages. GIs comprise a variety of MGEs subclasses, such as:

**(b) Prophage:** Bacteriophage are viruses of bacteria. Temperate phage integrate into the host genome and transmit vertically as prophage, in contrast to lytic phage that lyse the cell upon replication. A host is referred to as a lysogen if it carries one or more dormant prophage in its genome. Half of the available fully sequenced bacterial genomes were found to be lysogens (Touchon *et al*., 2016). Environmental or host signals trigger prophage to excise and enter the lytic life cycle, lysing the host and producing free viral particles to infect new replicons (Silpe *et al*., 2023).

**(c) Integrative and conjugative elements (ICEs)** and **integrative and mobilizable elements (IMEs):** GIs that can integrate into, and excise from, the host replicon (Audrey *et al*., 2023). These often integrate at conserved genes in the bacterial genome and show modular genetic architecture and divergent sequence composition (codon usage and GC content). Over half of sequenced bacteria contain ICEs, which encode conjugative transfer systems for horizontal transmission. By contrast, IMEs depend on other MGEs for horizontal transfer (Lang *et al*., 2025).

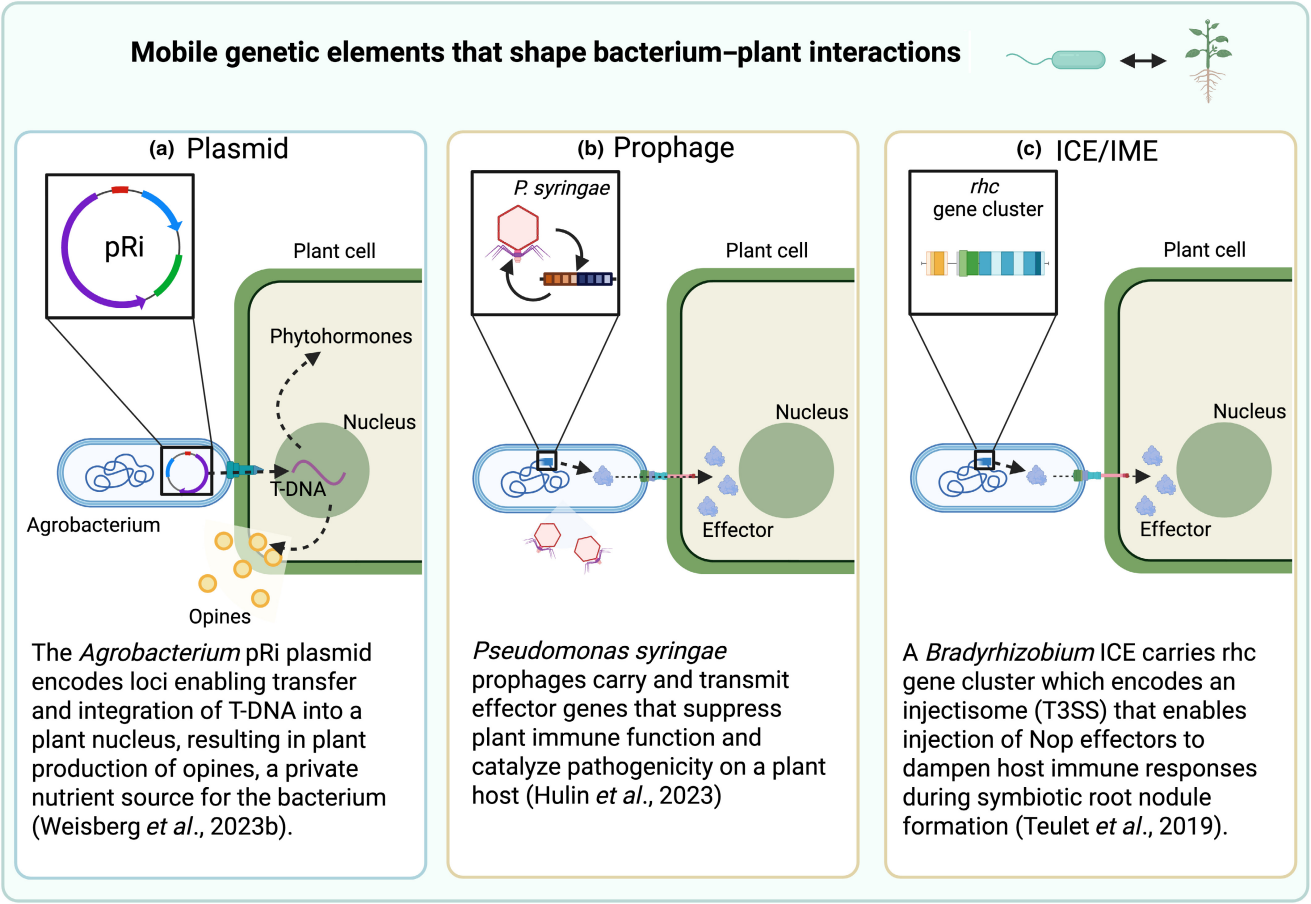

Box 1: Created in BioRender. Holtappels, D. (2025) https://BioRender.com/snso6o4.

## II. Fitness conflict and alignment during MGE‐mediated adaptation

Differentiating them from other loci, MGEs' potential for independent horizontal transmission causes them to have fitness interests distinct from their host replicon. This sets the stage for conflict because loci that increase MGE fitness, even at the cost of host replicon fitness, can be favored in MGEs, and vice versa (Brockhurst & Harrison, 2022). MGEs and the main replicon share fitness alignments as well: loci that increase the fitness of the cell they co‐inhabit can benefit both genomic compartments. These layers of fitness conflict and alignment may give rise to nested levels of adaptation that include loci for the MGE to benefit or exploit the host replicon or vice versa, and environmental adaptations that benefit the unified replicon (Batstone, 2022) (Box 2).

Vertical cotransmission aligns the fitness interests of an MGE and host replicon while horizontal transmission of MGEs via infection of novel replicons is initially costly to host replicon fitness (Brockhurst & Harrison, 2022). The resulting fitness conflict over MGE reproduction is epitomized by phage that kill their host but occurs to a lesser extent due to the costs of transfer of conjugative plasmids and integrative and conjugative elements (ICEs), which hijack the host replicon's replication machinery (Haudiquet *et al*., 2022). MGEs may initially capture cargo genes as an incidental by‐product of horizontal transmission (Vos *et al*., 2024). Subsequently, adaptive cargo genes may evolve under selection to benefit the shared bacterial cell and/or to benefit the MGE directly, such that the MGE bearing them sweeps to high frequency (Kehlet‐Delgado *et al*., 2024). Thus, MGEs are distinct from other loci that encode niche‐specific adaptations due to both their frequent horizontal transmission and their potential to fluctuate between evolving under fitness conflict and alignment with the host replicon.

**Fig. 1:**
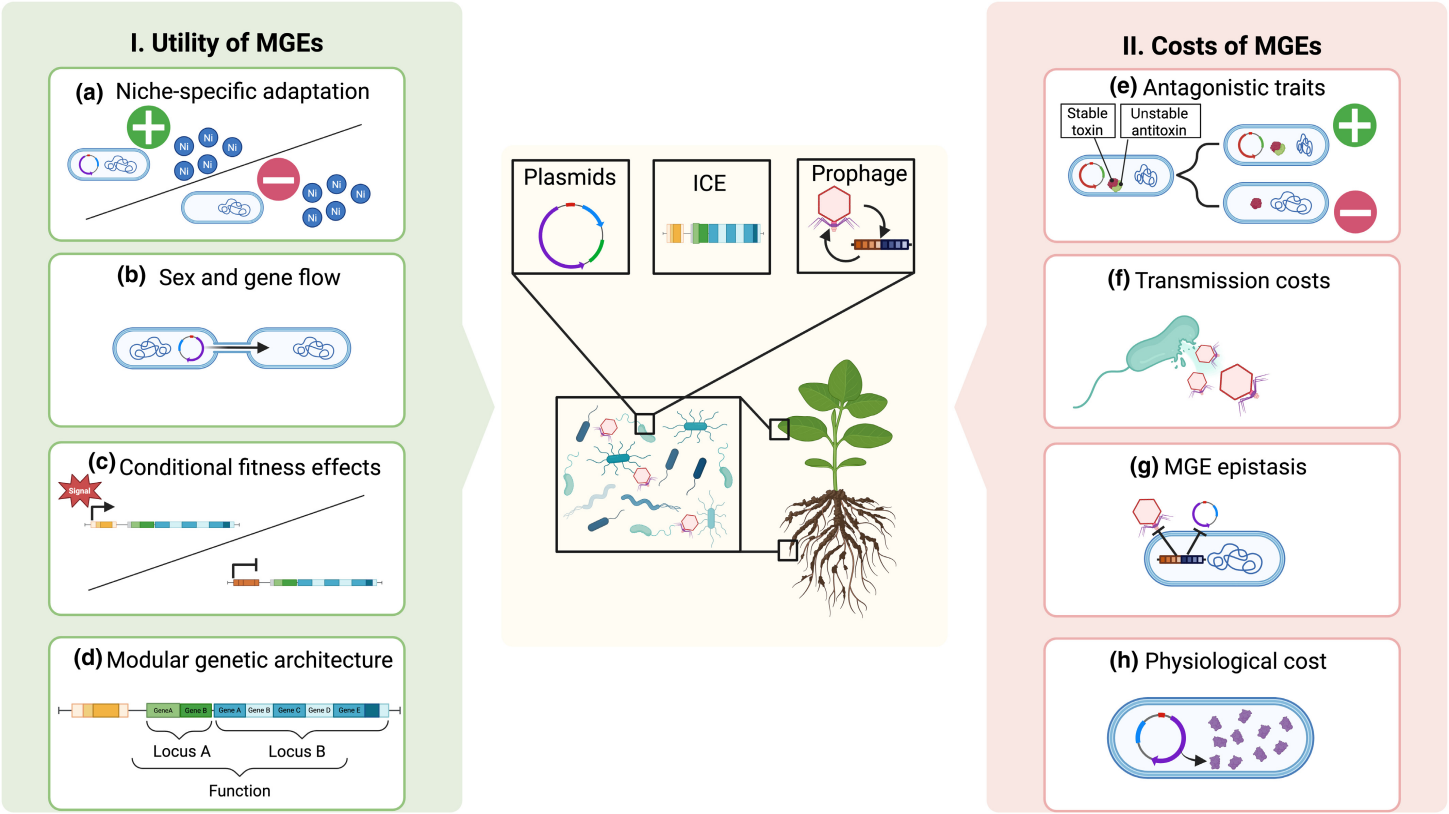
Conceptual overview of the costs and benefits of adaptation via loci carried by mobile genetic elements (MGEs). (I) MGEs can potentiate bacterial local adaptation by conferring the following. (a) Niche‐specific adaptive variants: cargo genes carried on MGEs are locally beneficial to bacteria. (b) Sex and gene flow: MGEs enable recombination and horizontal gene transfer among bacteria. (c) Conditional fitness effects: MGE gene expression and gain/loss may be adaptively plastic. (d) Modular genetic architecture: multiple, coadapted gene complexes underlying complex traits can be cotransmitted. II. MGE attributes that constrain their utility for bacterial local adaptation include the following. (e) Antagonistic traits: MGEs can acquire traits that benefit their own fitness at their host's expense. (f) Transmission costs: MGE transmission can harm or kill bacterial hosts. (g) MGE epistasis: some MGEs exclude infection by others, limiting the host's ability to adapt. (h) Physiological costs: MGE gene expression and replication can incur allocation and interference costs to bacterial hosts. See Sections IV and V for specific examples. Created in BioRender. Holtappels, D. (2025) https://BioRender.com/lnio3pw.

Given the potential fitness costs of exploitative MGEs, the prevalence of beneficial loci on MGEs poses an evolutionary dilemma: when do bacteria adapt to local conditions via loci on MGEs vs variants residing within the main replicon? To predict the prevalence of MGE‐borne loci as agents of local adaptation, and the cascading impacts on host plants, we provide a conceptual overview of the evolutionary utility of MGEs and then the evolutionary constraints they incur during bacterial adaptation (Fig. 1). Subsequently, we discuss specific examples of the evolutionary utility (Section IV) and constraints (Section V) of adaptation via MGEs in plant‐associated bacteria.

Box 2 **Mechanisms of transmission interference**

- Intracellular mechanisms that alter the mode and frequency of mobile genetic elements (MGE) spread will shift MGE‐replicon fitness conflict and alignment. If replicons curtail MGE horizontal transmission, this promotes fitness alignment. By contrast, if MGEs evolve to evade restrictions on their transfer, this enhances MGE horizontal transmission and potentiates fitness conflict with the main replicon. Below, numbers in the text link to examples in the figure.
**Plasmids**. The main replicon can interfere with plasmid reproduction by means of incompatibility (1), and defenses such as restriction enzymes and CRISPR Cas (Hülter *et al*., 2017). These mechanisms promote fitness alignment if they suppress entry of multiple MGEs into a cell and thus prevent a tragedy of the commons, whereby MGEs would compete for shared host resources, which could promote horizontal transmission. If the plasmid succeeds in evading these defenses (2) and highjacks the cell's replication machinery, the costs of plasmid reproduction, conjugation, and transmission (3) may select for new cellular defenses.
**Prophage**. The main replicon bears a plethora of antiprophage defenses (4). Prophage can be ‘domesticated’ within the main replicon if they acquire mutations or deletions that prevent initiation of the lytic stage (5) due to the high fitness costs of phage reproduction and transmission (lysis) to the main replicon (6). However, prophage will experience selection to escape domestication if horizontal transmission confers strong benefit to them.
**Integrative and conjugative elements (ICEs)/integrative and mobilizable elements (ICEs/IMEs)**. Quorum‐sensing molecules (7) can trigger excision of some ICEs (8) and may indicate conditions conducive to horizontal transmission to a naïve recipient. ICE excision increases conjugative transfer but can be repressed by regulation of quorum‐sensing signals produced by the ICE (9) and main replicon (10), shielding most cells from the costs of expression of ICE mobility genes (Wardell *et al*., 2021; Ramsay *et al*., 2022; Colombi *et al*., 2024).
- At the population and community level, MGEs and the diverse replicons they coevolve with experience heterogeneous conditions. Variable selection among replicons and environments shapes the evolutionary dynamics of MGE‐borne cargo genes that confer niche‐specific fitness.
- Overall, nested levels of adaptation emerge due to both intracellular fitness conflict/alignment and environmental adaptation: MGEs adapt to the local cellular environment, as well as the ecological niche in which the cell operates.

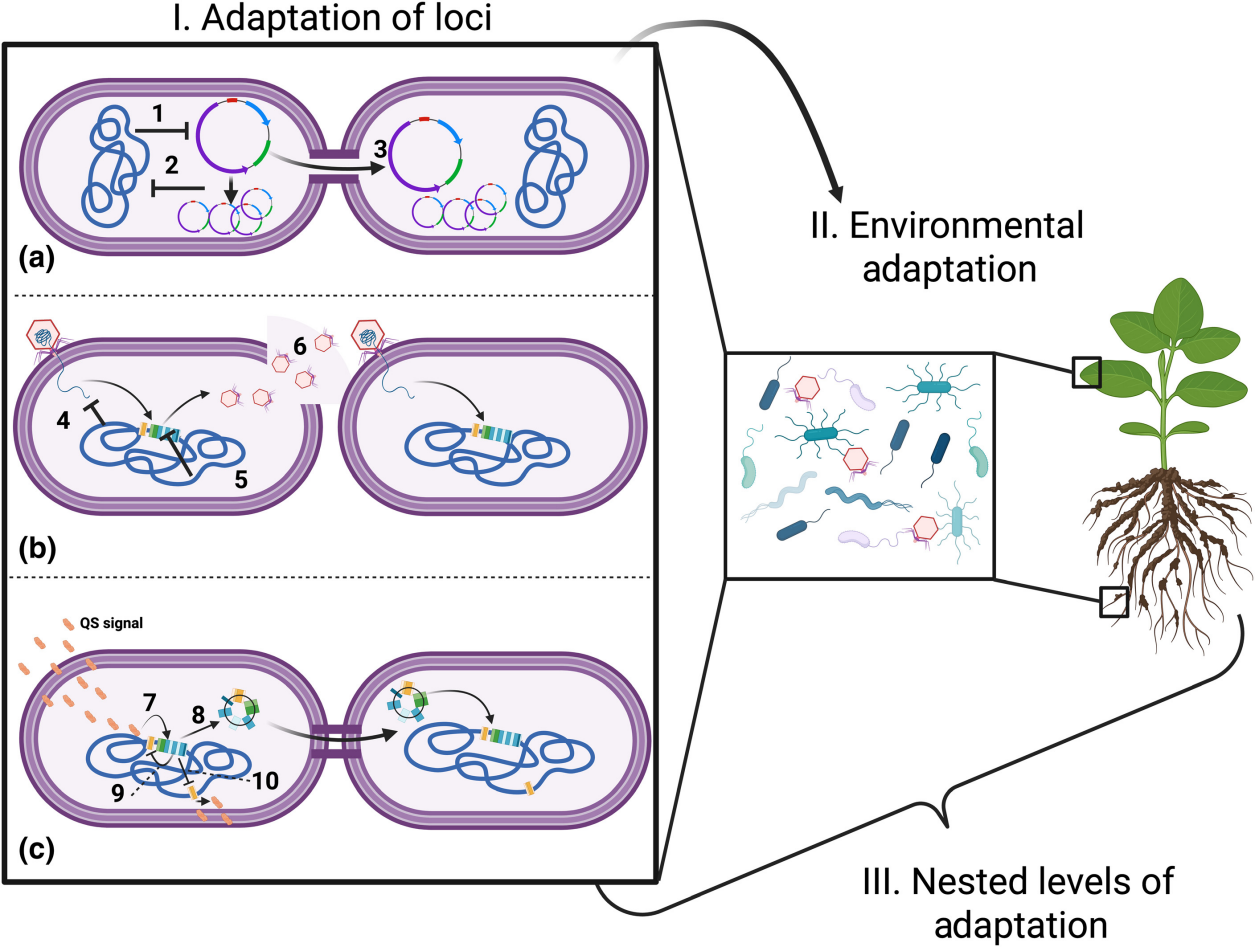

Box 2: Created in BioRender. Holtappels, D. (2025) https://BioRender.com/d8ysor6.

## III. The utility of MGEs for bacterial adaptation and consequences for host plants

### Cargo genes confer niche‐specific adaptation

The gain and loss of MGE‐associated cargo genes alters diverse adaptive bacterial phenotypes (Colombi *et al*., 2024; Vos *et al*., 2024). One common example is antibiotic resistance, in which pathogens such as *Erwinia amylovora*, which causes fire blight in apples and pears, can acquire plasmids carrying streptomycin resistance genes, locally reducing the effectiveness of antibiotic pesticides (Verhaegen *et al*., 2023). Similarly, Kehlet‐Delgado *et al*. (2024) find that symbiotic *Mesorhizobium* inhabiting serpentine soil outcrops enriched in toxic nickel acquire GIs that bear a nickel resistance *nre* operon. Acquisition of this GI drives parallel molecular solutions to nickel among populations across large spatial scales. Furthermore, cargo gene allelic variation may also fine‐tune local adaptation, as *nre* alleles confer contrasting levels of nickel resistance (Kehlet‐Delgado *et al*., 2024).

MGE cargo genes also orchestrate complex traits, such as intracellular symbiosis, which can underlie mosaics of adaptation between bacteria and biotic agents of selection. Non‐nodulating, free‐living rhizobia bacteria native to a soil can acquire sym ICE or plasmids carrying genes required for nitrogen‐fixing symbiosis (Colombi *et al*., 2023). This catalyzes a transformational evolutionary leap into nodulating, intracellular mutualists on novel hosts such as leguminous crops (Greenlon *et al*., 2019; Wardell *et al*., 2021). At a more nuanced level, rhizobia sym MGEs can diverge across the landscape into distinct, potentially locally adapted populations that interact with local plant genotypes, despite a lack of corresponding genetic structure for the main rhizobia replicon (Greenlon *et al*., 2019; Weisberg *et al*., 2022; Riaz *et al*., 2025). In plant pathogen *Pseudomonas syringae*, lambdoid prophages carry and disperse an arsenal of effector genes that shape virulence and host range (Holtappels *et al*., 2024). Ultimately, a bacterial chromosome could evolve to ‘capture’ locally adaptive cargo genes, allowing loss of the now redundant MGE (Brockhurst & Harrison, 2022). Understanding why MGE carriage of adaptive cargo genes persists is therefore critical to predict bacterial adaptation and impacts plant health.

### Horizontal transmission enables ‘sexual’ recombination and gene flow

Unlike vertically inherited bacterial loci, MGE‐encoded adaptive variants are shuffled by HGT among genomes to unlink beneficial and deleterious variants, potentiating adaptation by relieving clonal interference and rescuing beneficial mutations from deleterious backgrounds (Vos *et al*., 2024). In *Bacillus subtilis*, a common soil bacterium, HGT among strains via homologous recombination increases adaptation to elevated UV radiation by generating new adaptive combinations of donor and recipient loci, with up to 40% of observed HGT translocations under positive selection (Power *et al*., 2021). In rhizobia, sym ICE and plasmids are hotspots of recombination, structural rearrangement, mutation, and insertion–deletion. The resulting diversity of combinations of symbiosis loci provides variation upon which selection can act to shape host range and nitrogen fixation (Porter *et al*., 2018; Weisberg *et al*., 2022; Riaz *et al*., 2025). In plant pathogens such as *Pseudomonas syringae* pv *actinidiae*, conjugative ICEcu transfer can increase by orders of magnitude *in plantae*, potentiating adaptation via resistance to copper pesticides during crop production (Colombi *et al*., 2017). Furthermore, MGEs can incidentally transport bacterial replicon‐encoded loci horizontally among lineages and populations and thus impact recombination and gene flow genome‐wide (Haudiquet *et al*., 2022).

While introducing beneficial alleles to populations and replicons can increase adaptive evolution (Arnold *et al*., 2022), bacteria can also weaponize MGE transmission. Chromosomal compensatory mutations in *Pseudomonas fluorescens* not only offset the cost of plasmid carriage but also increase host competitive ability via transmission of these costly plasmids to naive competitors (Wright *et al*., 2024). Research comparing patterns of gene flow for cargo genes, MGEs, bacteria, and plant hosts will be critical to reveal how cargo genes arise and spread among plant‐associated microbes.

### Conditional fitness effects can minimize costs of MGE carriage

Cross‐environment fitness effects of MGEs are rarely measured yet are key to evolutionary dynamics. On the one hand, MGEs could be conditionally neutral, such that they are beneficial locally but have no effect on fitness elsewhere (Wadgymar *et al*., 2022). This could occur if MGE traits are inducibly expressed only when their benefits exceed their costs (Brockhurst & Harrison, 2022). In rhizobia, sym ICE or plasmid gene expression is induced by plant molecular signals during symbiosis, in which nitrogen fixation can accrue symbiotic fitness benefits (Liu *et al*., 2023). Likewise, expression of the *TN6212* element in *P. syringae* ICEs is triggered by preferred carbon sources produced by plants during pathogen establishment, contributing to disease progression (Colombi *et al*., 2024). These cues can be frequency‐dependent: adaptive genes in the ICEBs1 of plant growth promoting *B. subtilis* delay host cell biofilm and spore development to enhance growth but are only expressed and beneficial when the ICE is at a low frequency (Jones *et al*., 2021).

By contrast, MGEs could be antagonistically pleiotropic such that they are beneficial locally, but reduce fitness elsewhere (Wadgymar *et al*., 2022). Bacteria could evolve in response to this trade‐off such that lineages that lose an MGE are favored where it is costly, and those that acquire the MGE are favored if conditions shift such that it confers benefits (Carvalho *et al*., 2020). A more gradual MGE domestication can also occur whereby costly HGT genes are deleted, leaving only advantageous MGE remnants (Bobay *et al*., 2014). For example, tailocins are headless prophage‐tail remnants that function as bacterial weapons to kill competitors and shape plant pathogen diversity (Backman *et al*., 2024). Resolving MGE fitness effects across environments is critical to predict how shifting conditions affect bacterial fitness and MGE mobilization, with clear implications for plant health.

### Modular genetic architecture facilitates rapid evolution

MGEs can serve a function analogous to inversions or supergenes in plants, in that they can enable multiple adaptive genes and coadapted gene complexes to be co‐inherited and physically linked (Berdan *et al*., 2022). However, MGEs are distinct in that they can transmit independently of the host replicon. Coadapted loci and alleles underlying complex adaptations, such as pathogenesis or symbiotic mutualism, are often cotransmitted in a single MGE. *Agrobacterium* pathogenicity as crown gall or hairy root diseases depends on tumor‐inducing (pTi) or root‐inducing (pRi) oncogenic plasmids that carry hundreds of coadapted loci that must function in concert to invade and transform plant cells. These loci enable the pathogen to harness plant cells to produce particular opines, a private energy source if the pathogen cells carry compatible alleles for uptake and catabolism (Weisberg *et al*., 2023b). Thus, linked beneficial allele combinations on an MGE could enhance local adaptation when multitrait phenotypes are under divergent selection. Importantly, for MGEs to remain mobile, such coadapted gene complexes (and many epistatic alleles) must all be encoded within the MGE to provide consistent function to both the MGE and a host cell.

## IV. Costs of relying on MGEs for bacterial adaptation and consequences for plants

### MGEs carry traits antagonistic to the host

Traits that favor MGE fitness over that of the main replicon reduce the net fitness benefit of MGEs to bacteria that carry them. For example, systems that ensure high MGE copy number and those that mitigate MGE–MGE conflict are often costly to the host (Lang *et al*., 2025). Toxin–antitoxin gene drive systems epitomize costly antagonistic coevolutionary responses and lead to MGE ‘addiction’. MGEs can encode stable toxin and unstable antitoxin proteins such that in daughter cells that lack the MGE, the unstable antitoxin is not replenished, and toxin kills these daughters, enforcing plasmid maintenance (Jurėnas *et al*., 2022). Some toxin–antitoxin systems function as abortive infection systems, in which phage infection triggers a toxin that inhibits essential cellular functions, killing both phage and host (Aframian & Eldar, 2023). More broadly, when bacterial chromosomes capture conjugative plasmids, the resulting mobilizable plasmids are often lost over time (Coluzzi *et al*., 2022), consistent with the antagonistic costs of MGEs often exceeding their benefits to the host.

### MGE transmission imposes costs

Horizontal transmission increases MGE fitness at a cost to the host replicon, for example as a result of energetically expensive conjugative transfer (San Millan & MacLean, 2017; Bethke *et al*., 2022). This decreases net MGE fitness benefits to locally adapting bacteria. Moreover, MGEs can alter transmission rates based on environmental cues, which may optimize MGE fitness interests at the host's expense (Haudiquet *et al*., 2022) (Box 2). Quorum‐sensing molecules can stimulate conjugative transmission of sym elements and ICEBs1 when replicons lacking these elements are abundant in root‐associated microbiomes (Jones *et al*., 2021; Liu *et al*., 2023). Prophage can detect host condition and trigger induction in response to bacterial stress responses, which optimizes the production of infectious particles during stressful conditions, at the cost of bacterium lysis and death (Silpe *et al*., 2023). Phage can also use small molecules, such as the arbitrium system, to coordinate with progeny (and other MGEs) during infection to follow either the lytic or lysogenic cycle (Guler *et al*., 2024; Zamora‐Caballero *et al*., 2024). Whether transmission traits predict an MGE's role in bacterial local adaptation is not well‐understood, although MGEs with transmission mechanisms that lead to costly and high rates of HGT could play less of a role in host adaptation, as these traits decouple MGE fitness from that of a host cell.

### MGE epistasis constrains the bacterial adaptive landscape

The fitness burden arising from negative genetic interactions between chromosomal‐ and MGE‐encoded molecular machineries can be great enough to repeatedly select for compensatory mutations to reduce them. Such compensatory mutations occur for the bacterium, *P. fluorescens* during the acquisition of mercury resistance plasmids (Wright *et al*., 2024). Furthermore, the majority of over 150 known bacterial defense systems against MGEs are themselves MGE‐encoded, suggesting a benefit to the MGEs rather than, or in addition to, the host bacterium (Beamud *et al*., 2024). Epistatic effects in recipient cells, such as MGE superinfection exclusion, could limit the acquisition of multiple beneficial traits conferred by distinct MGEs within one cell (Lang *et al*., 2025), which could hinder bacterial adaptation. Furthermore, epistasis in the donor cell impacts the transfer of MGE‐borne adaptive loci, as occurs for mobilizable elements that require helper elements for horizontal transmission (Ares‐Arroyo *et al*., 2024). The IME TN1 in the phytopathogen *Streptomyces scabiei*, contains *txt* genes that encode phytotoxins, but these adaptive loci only transfer to a recipient if the donor also bears conjugation machinery from an ICE such as TN2 (Weisberg *et al*., 2023a). These examples highlight that epistatic effects are pervasive when multiple replicons unite in a single cell. Understanding how this epistasis constrains local adaptation in the phytomicrobiome is a promising frontier.

### MGEs impose physiological costs

Large MGEs can impose a metabolic burden and cytotoxicity that reduce growth rate and weaken competitiveness (Rodríguez‐Beltrán *et al*., 2021). In *Agrobacterium*, for example, virulence plasmids can be a metabolic drain to the cell, especially in low nutrient environments (Platt *et al*., 2011). Even compensatory mutations that mitigate MGE costs can themselves be costly (Wright *et al*., 2024). Thus, local adaptation can be constrained by the fact that bearing an MGE, even one containing adaptive loci, can be maladaptive in the face of competitors that lack it. Determining the spatial and temporal context of MGE physiological costs will provide insight into constraints on bacterial adaptation and host plant performance.

## V. Conclusion: impact of MGEs on plant health

MGE presence or absence can mean the difference between a bacterium acting as a pathogen, commensal, or mutualist to a plant (Colombi *et al*., 2023; Weisberg *et al*., 2023b). Given current reliance on 16S metabarcoding, which does not differentiate among ecotypes within taxons, as well as short‐read genome sequencing, which often does a poor job resolving larger plasmids, it is likely that we underestimate the ecological significance of MGE cargo genes. Ultimately, whether a locus encoding a trait that benefits or harms a plant exists on a microbial MGE or chromosome will critically alter its long‐term stability, evolutionary dynamics, and the spatial and temporal scale of adaptation. A better understanding of these MGE evolutionary dynamics is central to our ability to understand both the benefits of mutualists and the costs of antagonists for plant health. Moreover, designing novel microbial inocula and consortia to enhance plant health will require an expansion from a focus on microbial taxa to encompass the flexible complement of adaptive MGEs that underlie key microbial functions and traits (Box 3).

Box 3 **Future directions**

Local adaptation is common, yet we have a surprisingly limited understanding of the mechanisms that give rise to it (Wadgymar *et al*., 2022). To better predict and steer bacterial evolution in plant‐associated microbes, we highlight key unanswered questions regarding mobile genetic element (MGE) potentiation of, and constraints on, local adaptation. Both theoretical and empirical data are needed to resolve:

1. Do MGE characteristics, such as type or size, level of exploitation, transmission rate, or cotransmission with other MGEs, predict impacts on bacterial local adaptation and host plants?
2. What determines adaptive cargo gene migration across the landscape? What are the relative contributions of free‐living infectious replicons vs replicons integrated into a bacterial cell?
3. What is the relative importance of gain/loss of MGE cargo genes vs allelic variation among MGE cargo genes to local adaptation and host plant fitness?
4. How can natural genomic variation among lineages and populations and evolve‐and‐resequence experiments best be leveraged to reveal how diverse MGEs contribute to local adaptation in plant‐associated bacteria?
5. How do adaptive cargo genes become captured or arise within MGEs and spread within the plant microbiome? Why do they tend to confer traits that are locally beneficial rather than globally beneficial?
6. Is there conflict between MGEs and the main replicon over capture of adaptive loci, and can this lead to coevolution?
7. In what ways is current population genetic and quantitative genetic theory sufficient or insufficient to capture evolutionary dynamics of adaptive loci on MGEs?
8. Are there differences between the role of MGE in local adaptation in plant‐associated bacteria as compared to better studied human‐associates?
9. When might we need to consider the dangers of MGE‐mediated transmission of cargo genes when introducing microbial amendments or synthetic communities into natural systems?
10. How might the purposeful introduction of microbial taxa into plant systems be affected by the presence of MGEs within the natural microbial communities associated with that system?

## Competing interests

None declared.

## Author contributions

SP, DH, AM and BK contributed to the writing of the manuscript.

## Disclaimer

The New Phytologist Foundation remains neutral with regard to jurisdictional claims in maps and in any institutional affiliations.
